# Soil-transmitted helminthiasis in China: A national survey in 2014-2015

**DOI:** 10.1371/journal.pntd.0009710

**Published:** 2021-10-19

**Authors:** Ying-Dan Chen, Men-Bao Qian, Hui-Hui Zhu, Chang-Hai Zhou, Ting-Jun Zhu, Ji-Lei Huang, Zhong-Jie Li, Shi-Zhu Li, Xiao-Nong Zhou, Group on National Survey of Important Human Parasitic Diseases in China

**Affiliations:** 1 National Institute of Parasitic Diseases, Chinese Center for Disease Control and Prevention (Chinese Center for Tropical Diseases Research), Shanghai, China; 2 NHC Key Laboratory of Parasite and Vector Biology, Shanghai, China; 3 WHO Collaborating Center for Tropical Diseases, Shanghai, China; 4 National Center for International Research on Tropical Diseases, Shanghai, China; 5 School of Global Health, Chinese Center for Tropical Diseases Research, Shanghai Jiao Tong University School of Medicine, Shanghai, China; 6 Division of Infectious Disease, Key Laboratory of Surveillance and Early Warning on Infectious Disease, Chinese Centre for Disease Control and Prevention, Beijing, China; Federation University Australia, AUSTRALIA

## Abstract

**Background:**

Based on two national surveys, the prevalence of soil-transmitted helminthiasis (STH) in China had decreased from 53.58% in 1988–1992 to 19.56% in 2001–2004. To update the epidemiology and characteristics of STH in China, a third national survey was implemented in 2014–2015.

**Methodology/Principal findings:**

This survey covered rural areas in 31 provinces in mainland of China. Multiple-stage stratified cluster sampling was employed, which included three levels (provinces, ecozones and economical levels). Stool samples were collected and the Kato-Katz method was applied for helminth eggs detection. Samples with hookworm eggs were selected and hatched to differentiate the species based on larval morphology. Between June 2014 and May 2015, a total of 484,210 participants from 604 counties were enrolled. The weighted prevalence of STH overall was 4.49% (95% confidential interval (CI): 2.45%-6.53%), including 2.62% (95% CI: 0.86%-4.38%) hookworm infections, 1.36% (95% CI: 0.49%-2.23%) ascariasis, and 1.02% (95% CI: 0.15%-1.89%) trichuriasis. The estimated population infected was 29.12 million (95% CI: 15.88 million-42.32 million) for all STH; 16.97 million (95% CI: 5.57 million-28.39 million) for hookworm infections; 8.83 million (95% CI: 3.18 million-14.45 million) for ascariasis; and 6.60 million (95% CI: 0.97 million-12.25 million) for trichuriasis. Overall, the prevalence of ascariasis and trichuriasis was relatively high in children, while hookworm infections were more common in the older population, especially those over 60. STH was highly prevalent in western China, and moderately in central areas, but low in eastern and northern regions. Out of 3,579 hookworm cases with species differentiation, 479 cases (13.38%) were infected with only *Ancylostoma* spp., 2,808 cases (78.46%) with only *Necator americanus*, and another 292 cases (8.16%) with both species.

**Conclusions/Significance:**

This survey demonstrated the continuous decrease of STH in rural China. However, endemicity still prevails in the western areas of the country. Hookworm, especially *N*. *americanus*, is becoming the predominant species. Older farmers in western China should be prioritized for control due to the high prevalence of hookworm.

## Author summary

Soil-transmitted helminthiasis (STH) had decreased significantly in China based on the two national surveys implemented in 1988–1992 and 2001–2004, respectively. In 2014–2015, an updated national survey on STH was implemented in rural China. This survey covered rural areas of 31 provinces in mainland of China and multiple-stage stratified cluster sampling was employed. Stool samples were collected and examined for helminth eggs. Totally, 484,210 villagers from 604 counties in 31 provinces participated in the survey. The adjusted prevalence of STH was 4.49%, and that of hookworm infections, ascariasis, trichuriasis was 2.62%, 1.36%, 1.02%, respectively. The estimated population with STH was 29.12 million, while that with hookworm infections, ascariasis, trichuriasis was 16.97 million, 8.83 million, 6.60 million, respectively. Children showed a high prevalence in ascariasis and trichuriasis, while the older population had a high prevalence of hookworm infections. STH was still highly prevalent in western parts, moderately in central areas, and low in eastern and northern regions. Further efforts are needed to control STH in China.

## Introduction

Soil-transmitted helminthiasis (STH) constitutes a group of helminth diseases caused by *Ascaris lumbricoides*, *Trichuris trichiura* and hookworm (*Ancylostoma* spp. and *Necator americanus*), which still imposes a significant health burden [1,2]. Of the neglected tropical diseases, STH are the most prevalent, producing a total of 3.38 million disability-adjusted life years in 2015, out of which ascariasis, trichuriasis and hookworm infections contributed 1.08 million, 0.54 million and 1.76 million, respectively [3]. Due to continuing transmission favoured by the predominant tropical environment and the still low social and economic development, their prevalence in Asia, Africa and Latin America remains high [4–6]. Related to the productive activities, adults are usually highly susceptible to hookworm infections, while *A*. *lumbricoides* and *T*. *trichiura* are more common in children [4–6].

STH used to be a widespread, serious threat to public health in China. Two national surveys of intestinal helminthiasis using the Kato-Katz method were carried out in mainland of China in 1988–1992 and 2001–2004 [7,8]. The results of the first survey, showed that altogether 646 million were infected with prevalence rates of all STH, ascariasis, trichuriasis and hookworm infections amounting to 53.58%, 47.00%, 18.80% and 17.17%, respectively [7]. At the time of the second survey, the corresponding prevalence rates had declined to 19.56%, 12.72%, 4.63%, and 6.12%, with the total cases estimated at about 129 million [8]. Due to the difference in natural environment as well as economic development, STH is more common in western and southern China [7,8]. Spot checks carried out between 2006 and 2013 have established a continued general decline of STH from 20.88% to 3.12% [9,10]. To capture the updated national epidemiology of these infections, a third national survey to guide the final strategy and steps of the national STH control programme was implemented in rural China in 2014–2015.

## Material and methods

### Ethics statement

The study was approved by the ethics committees in the National Institute of Parasitic Diseases at Chinese Center for Disease Control and Prevention. The objectives, procedures and potential risks of this study were orally explained and informed to all participants. A written consent form was also obtained with signature of the participant or his/her guardian for a child.

### Study design

This was a national cross-sectional sampling survey, covering 31 provinces in mainland of China. In consideration of the extreme low prevalence of STH in urban areas owing to high coverage of clean water and sanitation, only rural areas were covered for STH in this survey. A multiple-stage stratified cluster sampling strategy was employed, involving with three levels (provinces, ecozones and economic levels).

### Sampling

The province level was treated as the main stratum followed by all types of ecozones in each province as the 1^st^ substratum. The ecozones were defined based on type and natural environment according to the *Ecozone Classification in China* [11]. An ecozone usually covers several neighboring provinces and a province usually contains several different types of ecozones. Nationally, 50 ecozones are classified, but we included only 46 of them while the other four with very limited population were combined into neighboring ones. The sampling size was calculated for each 1^st^ substratum using binomial distribution as well as the expected prevalence data. The expected prevalence referred to the data from a post-survey spot checks (in 22 selected provinces carried out in 2012). An expected prevalence of 1% was set in the other nine provinces where the endemic level of infection was perceived as low although not backed by actual surveillance data. Additionally, the same expected prevalence was set for all of 1^st^ substrata in the same province. An allowance error was set at 10%, 15% and 30% when the expected prevalence was > 20%, 5%-20% and < 5%, respectively. Finally, an increase of 50% was used for the sample size.

Below the 1^st^ substratum level, the counties made up the 2^nd^ substratum. They were classified into three levels based on the economic level as expressed by the rural net income per capita, which was provided by the local economic departments. According to the share of total rural population in the 1^st^ substratum, the sampling size was distributed proportionally with respect to the 2^nd^ substratum. The sampling unit was set as the natural village, i.e. a rural collection of houses comprising about 250 villagers, and the number of sampling units in each of the 2^nd^ substratum as calculated. Usually, 3 units were included in each selected county. If the number was not integral, 2 or 4 units were selected. Thus, the number of counties needed to be sampled was decided there and then. In each sampled county, all towns were classified into 2–4 groups based on economic and natural conditions, then one town was sampled from each group. As a rural town is usually made up by dozens of natural villages, one natural village was sampled from each town.

### Procedure

Villagers were invited to provide a fresh stool sample (about 30 g) in a container. Stool samples were transferred to local county-level Center for Disease Control and Prevention (CDC) or Control Station of Parasitic Diseases. From each stool sample, double Kato-Katz thick smears [12], using 41.7 mg templates, were prepared and then examined qualitatively and quantitatively for helminth eggs under a microscope by trained technicians. The preparation of Kato-Katz smears was usually done within 24 hours after samples were collected. Smears were examined within 30 minutes after preparation to avoid the disappearance of hookworm eggs.

In each sampled county, 50 individuals with confirmed hookworm infection by the Kato-Katz method were sampled and their stool was further processed to differentiate between the hookworm species by the test-tube filter-paper hatching method [12]. If the number of cases with hookworm infections by the Kato-Katz method was less than 50, they were all included. In brief, 0.5 g of fresh stool were placed on a folded strip of filter paper (9.0 cm in length and 1.6 cm in width), which was then placed in a plastic tube (11.5 cm in length and 1.5 cm in width) containing 2 ml of sterile distilled water and incubated at 30°C for 4–5 days. Then, filariform larvae were taken to differentiate the species by the morphology, especially the differences of the oral spear and transverse lines on the tunica vaginalis [12]. All larvae were differentiated if the total number was below 100 in single sample, while the number exceed 100, only 100 larvae were examined.

### Statistical analysis

Individual egg counts, expressed as eggs per gram (EPG) of stool, were calculated by multiplying the sum of the egg counts in the 41.7 mg template used by the two Kato-Katz thick smears by 12. Infection intensity was categorized as per WHO guideline [13]. In hookworm infections, it was categorized as light (1–1999 EPG), moderate (2000–3999 EPG), and heavy (≥ 4000 EPG); in ascariasis, the categories contained light (1–4999 EPG), moderate (5000–49999 EPG), and heavy (≥ 50000 EPG); in trichuriasis, the categories contained light (1–999 EPG), moderate (1000–9999 EPG), and heavy (≥ 10000 EPG). Ages were classified into groups with an interval of 5 years, except those aged over 85 who were considered as a single group.

Weighted prevalence was applied. To calculate this, the weight (*W*_*i*_) was firstly captured, which was integrated with the base sampling weight (*W*_*base*_) and post-sampling adjusted weight (*W*_*adj*_) as expressed by:

$$w_{i}=w_{base}\times w_{adj}$$ (Eq 1)

where *W*_*adj*_ is the ratio of the total population of the age and gender group divided by the sampled population in the same group. The total rural population figure was extracted from the sixth national population census in 2010. *W*_*base*_ is the multiplied sampling weights in the three strata as expressed by:

$$w_{base}=w_{1}\times w_{2|1}\times w_{3|2|1}$$ (Eq 2)

where *W*_*1*_ is the weight in sampling county in each 2^nd^ substratum, *W*_*2|1*_ the weight of the sampled town in each sampled county and *W*_*3|2|1*_ the weight of the sampled natural villages of each sampled town. The weighted prevalence, p^, in the total rural population was calculated based on follows:

p^=∑i∈Swiyi∑i∈Swi (Eq 3)

where *w*_*i*_ is described by Eq 1, *y*_*i*_ the result of stool examination of the *i*^th^ individual and *S* the sample size. The total population infected, *n*, was calculated by:

$$n=\sum_{i\in S}w_{i}y_{i}$$ (Eq 4)

Taylor Series Expansion Method was employed to estimate the confidential interval (CI). Data were presented both at the province and ecozone levels, for each species, as well as the overall group of STH. The proportion of two genera of human hookworm, namely *Ancylostoma* spp. and *N*. *americanus* was also presented.

## Results

### Characteristics of participants

The survey took place between June 2014 and May 2015 and included 484,210 participants in total (238,505 males and 245,705 females) and they came from 1,890 sampling units in 604 counties belonging to 31 provinces. Among the sampled population, 5,423 persons (1.12%) were found to be infected with hookworm, 4,343 (0.90%) with *A*. *lumbricoides*, 1,756 (0.36%) with *T*. *trichiura*. Overall, 10,681 (2.21%) of them were infected with at least one helminth species.

### STH prevalence

**Fig 1:**
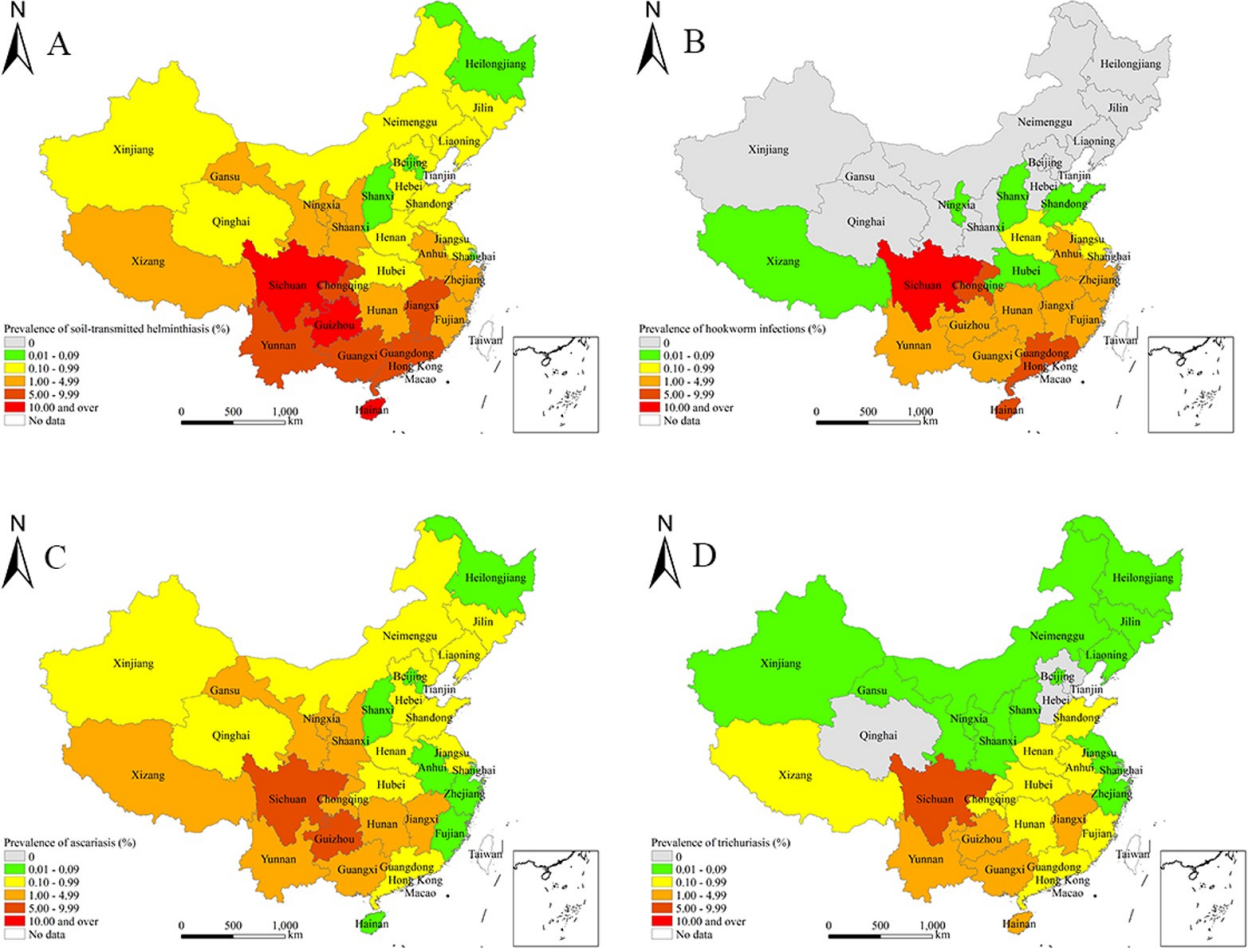
Weighted prevalence of soil-transmitted helminthiasis by provinces in China in 2014–2015. A. Soil-transmitted helminthiasis. B. Hookworm infections. C. Ascariasis. D. Trichuriasis. The base layer is from https://www.webmap.cn/mapDataAction.do?method=forw&resType=5&storeId=2&storeName=%E5%9B%BD%E5%AE%B6%E5%9F%BA%E7%A1%80%E5%9C%B0%E7%90%86%E4%BF%A1%E6%81%AF%E4%B8%AD%E5%BF%83&fileId=BA420C422A254198BAA5ABAB9CAAFBC1 with credit to National Catalogue Service For Geographic Information.

**Fig 2:**
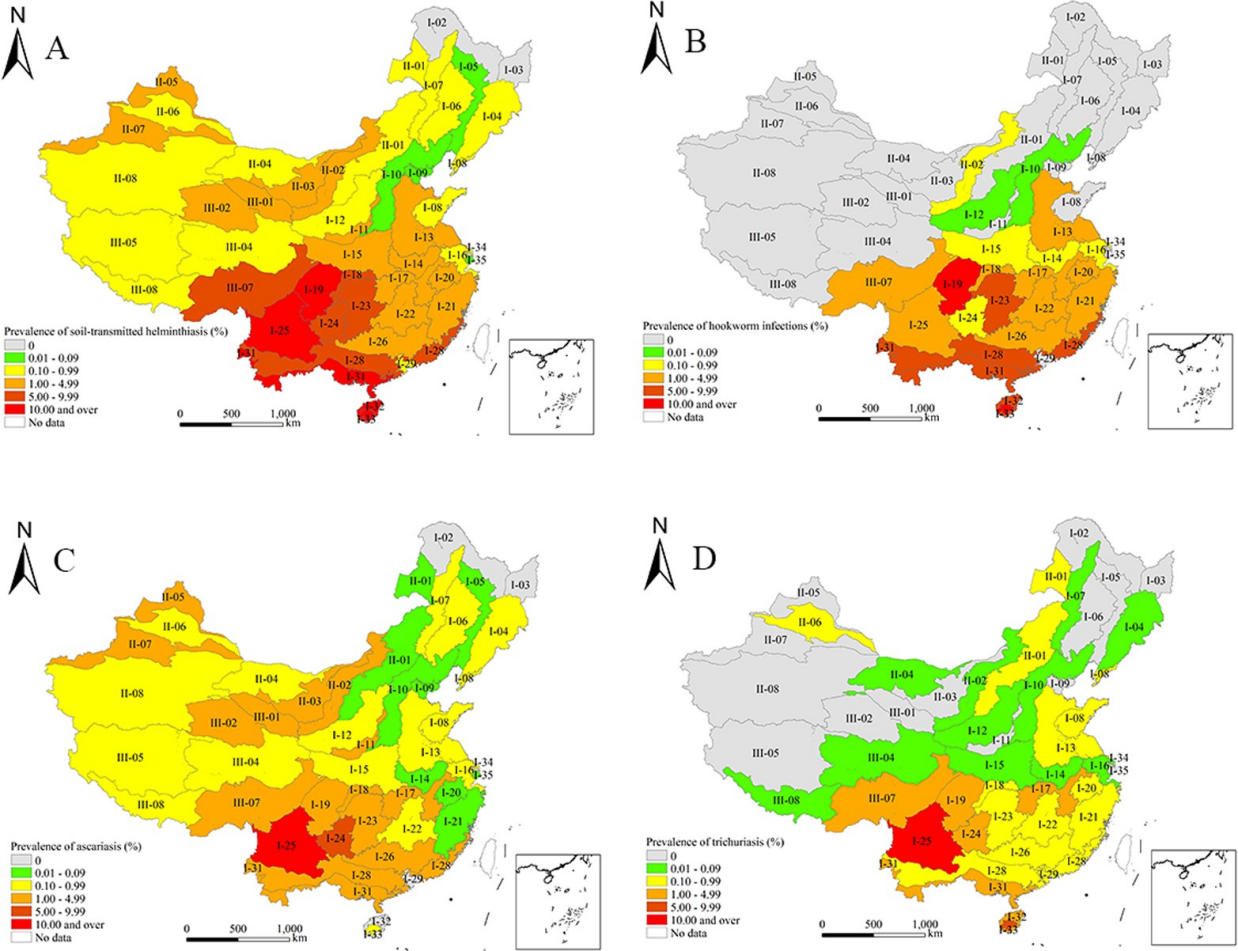
Weighted prevalence of soil-transmitted helminthiasis by ecozones in China in 2014–2015. A. Soil-transmitted helminthiasis. B. Hookworm infections. C. Ascariasis. D. Trichuriasis. The base layer is from https://www.webmap.cn/mapDataAction.do?method=forw&resType=5&storeId=2&storeName=%E5%9B%BD%E5%AE%B6%E5%9F%BA%E7%A1%80%E5%9C%B0%E7%90%86%E4%BF%A1%E6%81%AF%E4%B8%AD%E5%BF%83&fileId=BA420C422A254198BAA5ABAB9CAAFBC1 with credit to National Catalogue Service For Geographic Information.

A weighted national prevalence of 4.49% (95% CI: 2.45%-6.53%) was demonstrated for STH (**Tables 1** and **S1**). STH were detected in all 31 provinces, of which nine exceeded the average. The highest prevalence was found in Sichuan province (23.55%, 95% CI: 12.11%-34.99%), followed by Hainan (12.23%, 95% CI: 9.16%-15.30%) and Guizhou (10.68%, 95% CI: 7.38%-13.97%) (**Table 1** and **Fig 1A**). STH were found in 44 out of 46 ecozones, of which 10 exceed the average. The prevalence was highest in the Sichuan Basin ecozone (22.16%; 95% CI: 10.84%-33.48%), followed by Hainan Central Mountain ecozone (21.92%; 95% CI: 17.52%-26.33%) (**S1 Table** and **Fig 2A**). Thus, STH was still highly prevalent (> 5%) in the western regions and moderately so (> 1%) in the eastern and central areas, while the northern areas showed a low prevalence.

**Table 1:** Weighted prevalence and estimated population infected of soil-transmitted helminthiasis by provinces in China in 2014–2015.

Province	No. sampled	No. infected	Prevalence (%)	Weighted prevalence (%) (95% CI)	Estimated population infected
**Beijing**	13401	12	0.09	0.05 (0.00–0.13)	1419 (0–3446)
**Tianjin**	11107	7	0.06	0.03 (0.00–0.07)	690 (0–1792)
**Hebei**	26526	19	0.07	0.16 (0.00–0.32)	63307 (0–128125)
**Shanxi**	19500	3	0.02	0.00 (0.00–0.01)	801 (0–1828)
**Neimenggu**	20132	23	0.11	0.19 (0.02–0.36)	19941 (2065–37178)
**Liaoning**	26520	78	0.29	0.27 (0.01–0.52)	43749 (1637–85144)
**Jilin**	19683	74	0.38	0.36 (0.00–0.74)	43968 (0–89466)
**Heilongjiang**	26456	3	0.01	0.00 (0.00–0.01)	462 (0–1549)
**Shanghai**	13547	13	0.10	0.04 (0.00–0.11)	1025 (0–2653)
**Jiangsu**	13275	35	0.26	0.24 (0.14–0.34)	74879 (43303–105165)
**Zhejiang**	19935	387	1.94	1.38 (0.48–2.27)	288066 (100448–475034)
**Anhui**	12300	291	2.37	4.48 (0.76–8.20)	1377543 (233690–2521397)
**Fujian**	7721	209	2.71	1.58 (0.86–2.31)	238786 (129614–348148)
**Jiangxi**	18649	1219	6.54	5.61 (4.01–7.22)	1380461 (986690–1776534)
**Shandong**	13620	100	0.73	0.63 (0.00–1.35)	301081 (0–648317)
**Henan**	26866	99	0.37	0.47 (0.00–1.09)	269771 (0–630264)
**Hubei**	9215	118	1.28	0.45 (0.09–0.81)	122741 (24523–220704)
**Hunan**	26389	1140	4.32	4.60 (2.84–6.37)	1630143 (1005684–2255707)
**Guangdong**	9309	322	3.46	6.37 (1.04–11.70)	2051225 (335012–3768882)
**Guangxi**	5702	348	6.10	7.07 (4.28–9.85)	1862301 (1128112–2596238)
**Hainan**	2698	390	14.46	12.23 (9.16–15.30)	523656 (392265–655203)
**Chongqing**	9250	662	7.16	7.90 (6.20–9.60)	975358 (765461–1185229)
**Sichuan**	11403	1488	13.05	23.55 (12.11–34.99)	12057181 (6199625–17912872)
**Guizhou**	7572	1127	14.88	10.68 (7.38–13.97)	2400557 (1659200–3140790)
**Yunnan**	5067	950	18.75	8.73 (3.38–14.08)	2596412 (1004966–4186366)
**Xizang**	17939	268	1.49	1.61 (0.17–3.05)	33177 (3500–62790)
**Shaanxi**	19900	209	1.05	1.57 (0.21–2.94)	322025 (42938–601134)
**Gansu**	17437	335	1.92	1.62 (0.72–2.52)	284791 (126722–443527)
**Qinghai**	12859	138	1.07	0.91 (0.43–1.39)	27596 (13016–42075)
**Ningxia**	13346	303	2.27	1.94 (0.96–2.93)	63284 (31255–95393)
**Xinjiang**	26886	311	1.16	0.51 (0.10–0.93)	61614 (12057–112126)
**Total**	484210	10681	2.21	4.49 (2.45–6.53)	29118009 (15877565–42318571)

#### Hookworm infections

Hookworm infections had a weighted prevalence of 2.62% (95% CI: 0.86%-4.38%) nationally (**Tables 2** and **S2**). Hookworm infections were detected in 19 provinces, of which 10 exceeded the average. The highest prevalence was found in Sichuan province (14.55%, 95% CI: 1.79%-27.30%), followed by Hainan (8.10%, 95% CI: 3.61%-12.59%) (**Table 2** and **Fig 1B**). Hookworm infections were found in 22 out of 46 ecozones, of which nine exceed the average. The highest weighted prevalence was found in Sichuan Basin ecozone (20.34%; 95% CI: 8.27%-32.41%), followed by Hainan Central Mountain ecozone (15.54%; 95% CI: 9.97%-21.10%) (**S2 Table** and **Fig 2B**). Overall, hookworm infections were extremely common in the western regions, moderately so in the southern areas, while the northern areas were non-endemic.

**Table 2:** Weighted prevalence and estimated population infected of hookworm by provinces in China in 2014–2015.

Province	No. sampled	No. infected	Prevalence (%)	Weighted prevalence (%) (95% CI)	Estimated population infected (95% CI)
**Beijing**	13401	0	0.00	0.00	0
**Tianjin**	11107	0	0.00	0.00	0
**Hebei**	26526	0	0.00	0.00	0
**Shanxi**	19500	1	0.01	0.00 (0.00–0.00)^a^	196 (0–585)
**Neimenggu**	20132	0	0.00	0.00	0
**Liaoning**	26520	0	0.00	0.00	0
**Jilin**	19683	0	0.00	0.00	0
**Heilongjiang**	26456	0	0.00	0.00	0
**Shanghai**	13547	0	0.00	0.00	0
**Jiangsu**	13275	18	0.14	0.11 (0.02–0.20)	34360 (6186–61862)
**Zhejiang**	19935	357	1.79	1.30 (0.45–2.16)	272393 (94170–452015)
**Anhui**	12300	259	2.11	4.21 (0.62–7.81)	1295564 (190642–2401477)
**Fujian**	7721	186	2.41	1.45 (0.76–2.14)	218171 (114542–322527)
**Jiangxi**	18649	935	5.01	3.49 (2.26–4.72)	858162 (556089–1161390)
**Shandong**	13620	3	0.02	0.01 (0.00–0.03)	4362 (0–14407)
**Henan**	26866	28	0.10	0.11 (0.00–0.30)	62757 (0–173467)
**Hubei**	9215	52	0.56	0.06 (0.00–0.19)	16485 (0–51770)
**Hunan**	26389	701	2.66	2.71 (1.34–4.08)	959555 (474513–1444786)
**Guangdong**	9309	225	2.42	5.47 (0.48–10.45)	1760943 (154621–3366224)
**Guangxi**	5702	209	3.67	3.79 (2.90–4.69)	999527 (764375–1236178)
**Hainan**	2698	225	8.34	8.10 (3.61–12.59)	346769 (154594–539150)
**Chongqing**	9250	468	5.06	5.67 (3.69–7.66)	700516 (455573–945714)
**Sichuan**	11403	811	7.11	14.55 (1.79–27.30)	7446876 (916377–13976033)
**Guizhou**	7572	477	6.30	3.81 (0.14–7.48)	856512 (31475–1681683)
**Yunnan**	5067	457	9.02	3.83 (0.66–7.00)	1138563 (196236–2081290)
**Xizang**	17939	4	0.02	0.02 (0.00–0.04)	369 (0–823)
**Shaanxi**	19900	0	0.00	0.00	0
**Gansu**	17437	0	0.00	0.00	0
**Qinghai**	12859	0	0.00	0.00	0
**Ningxia**	13346	7	0.05	0.08 (0.00–0.18)	2443 (0–5860)
**Xinjiang**	26886	0	0.00	0.00	0
**Total**	484210	5423	1.12	2.62 (0.86–4.38)	16974524 (5573349–28385198)

^a^ 0.0011 (0.0000–0.0032)

**Fig 3:**
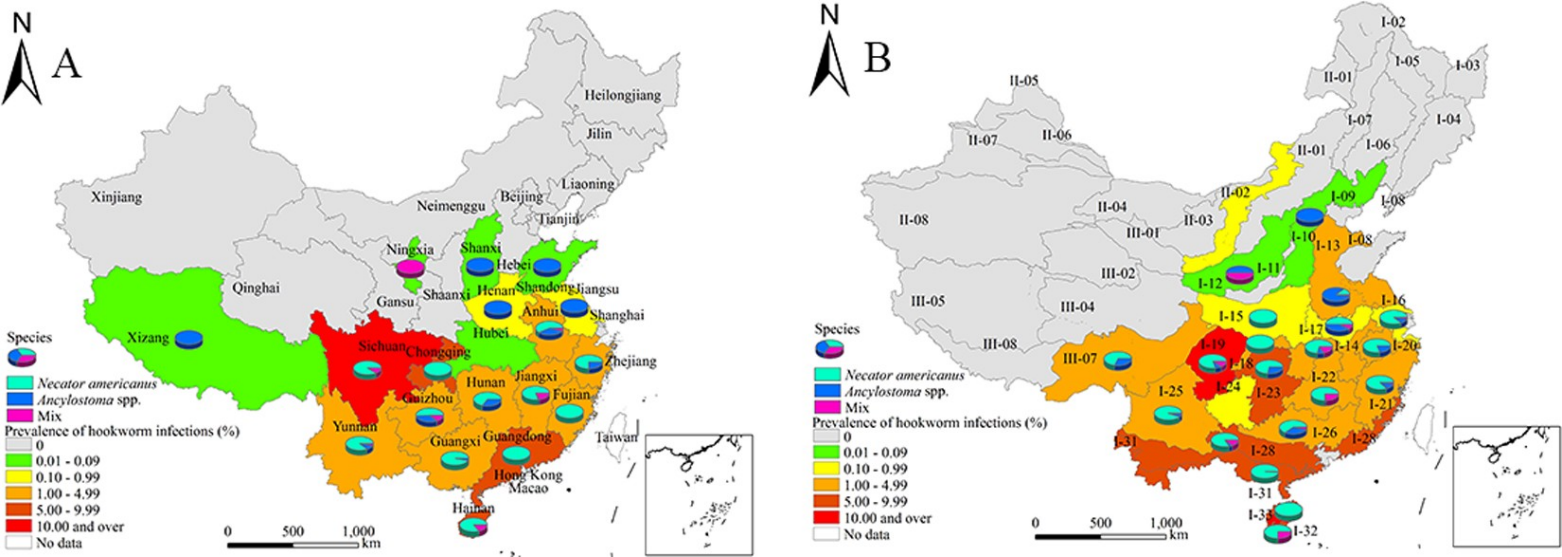
The proportion of hookworm species in China in 2014–2015. A. By provinces. B. By ecozones. The base layer is from https://www.webmap.cn/mapDataAction.do?method=forw&resType=5&storeId=2&storeName=%E5%9B%BD%E5%AE%B6%E5%9F%BA%E7%A1%80%E5%9C%B0%E7%90%86%E4%BF%A1%E6%81%AF%E4%B8%AD%E5%BF%83&fileId=BA420C422A254198BAA5ABAB9CAAFBC1 with credit to National Catalogue Service For Geographic Information.

Out of the overall 5,423 cases with hookworm infections identified by the Kato-Katz method, 3,579 were subjected to species differentiation. Among them, 479 (13.38%) cases were infected with only *Ancylostoma* species, 2,808 (78.46%) with only *N*. *americanus*, while 292 (8.16%) cases were simultaneously infected with both species. At the provincial level, 19 showed infection with at least one hookworm species, and out of the 18 where species differentiation was attempted, five showed only *Ancylostoma* species infections, two only *N*. *americanus* infections, while another 11 had both (**Fig 3A**). Out of 22 ecozones with hookworm infections, species differentiation was performed on 20. One ecozone was detected with only *Ancylostoma* species infections, three with only *N*. *americanus* infections and 16 ecozones had both hookworm genera (**Fig 3B**). Overall, *Ancylostoma* spp. was predominantly endemic in the northern areas, while *N*. *americanus* were mainly found in the southern regions.

#### Ascariasis

Ascariasis showed a weighted prevalence of 1.36% (95% CI: 0.49%-2.23%) nationally (**Tables 3** and **S3**). Ascariasis were detected in all 31 provinces, out of which nine exceeded the average. The highest prevalence was found in Sichuan province (6.83%, 95% CI: 0.00%-17.45%), followed by Guizhou (6.15%, 95% CI: 3.59%-8.71%) (**Table 3** and **Fig 1C**). Ascariasis was found in 42 of 46 ecozones, out of which 10 exceed the average. The highest weighted prevalence was found in South Western Sichuan—Central Northern Yunnan Mountain ecozone (10.02%; 95% CI: 0.00%-24.57%), followed by Central Guizhou Karst ecozone (5.30%; 95% CI: 3.68%-6.93%) (**S3 Table** and **Fig 2C**). Overall, ascariasis was highly prevalent (> 5%) in the western regions but less prevalent in the northern and eastern areas.

**Table 3:** Weighted prevalence and estimated population infected of ascariasis by provinces in China in 2014–2015.

Province	No. sampled	No. infected	Prevalence (%)	Weighted prevalence (%) (95% CI)	Estimated population infected (95% CI)
**Beijing**	13401	12	0.09	0.05 (0.00–0.13)	1419 (0–3446)
**Tianjin**	11107	7	0.06	0.03 (0.00–0.07)	690 (0–1792)
**Hebei**	26526	19	0.07	0.16 (0.00–0.32)	63307 (0–128125)
**Shanxi**	19500	1	0.01	0.00 (0.00–0.00)^a^	97 (0–292)
**Neimenggu**	20132	18	0.09	0.11 (0.00–0.26)	11741 (0–26851)
**Liaoning**	26520	76	0.29	0.26 (0.00–0.51)	42088 (0–83507)
**Jilin**	19683	72	0.37	0.35 (0.00–0.72)	42293 (0–87048)
**Heilongjiang**	26456	2	0.01	0.00 (0.00–0.00)^b^	222 (0–666)
**Shanghai**	13547	11	0.08	0.04 (0.00–0.09)	899 (0–2171)
**Jiangsu**	13275	16	0.12	0.12 (0.08–0.16)	37687 (24745–49490)
**Zhejiang**	19935	13	0.07	0.04 (0.00–0.07)	7488 (0–14649)
**Anhui**	12300	12	0.10	0.06 (0.00–0.12)	17698 (0–36898)
**Fujian**	7721	4	0.05	0.03 (0.00–0.08)	5229 (0–12057)
**Jiangxi**	18649	162	0.87	1.25 (0.43–2.08)	308656 (105805–511799)
**Shandong**	13620	22	0.16	0.12 (0.00–0.24)	57591 (0–115256)
**Henan**	26866	50	0.19	0.21 (0.00–0.50)	119778 (0–289112)
**Hubei**	9215	40	0.43	0.14 (0.00–0.29)	38630 (0–79017)
**Hunan**	26389	428	1.62	1.93 (0.96–2.91)	685100 (339950–1030472)
**Guangdong**	9309	63	0.68	0.81 (0.23–1.38)	260207 (74089–444535)
**Guangxi**	5702	76	1.33	1.93 (0.94–2.92)	508485 (247763–769646)
**Hainan**	2698	2	0.07	0.02 (0.00–0.07)	933 (0–2998)
**Chongqing**	9250	211	2.28	2.48 (1.07–3.90)	306227 (132104–481499)
**Sichuan**	11403	540	4.74	6.83 (0.00–17.45)	3495270 (0–8933398)
**Guizhou**	7572	614	8.11	6.15 (3.59–8.71)	1382450 (807118–1958216)
**Yunnan**	5067	381	7.52	2.19 (0.54–3.84)	651399 (160557–1141736)
**Xizang**	17939	216	1.20	1.35 (0.13–2.58)	27885 (2676–53114)
**Shaanxi**	19900	209	1.05	1.57 (0.21–2.94)	322025 (42938–601134)
**Gansu**	17437	333	1.91	1.62 (0.72–2.51)	284263 (126722–441767)
**Qinghai**	12859	138	1.07	0.91 (0.43–1.39)	27596 (13016–42075)
**Ningxia**	13346	289	2.17	1.82 (0.82–2.82)	59253 (26697–91812)
**Xinjiang**	26886	306	1.14	0.49 (0.08–0.91)	59566 (9645–109714)
**Total**	484210	4343	0.90	1.36 (0.49–2.23)	8826171 (3175513–14451824)

^a^ 0.0005 (0.0000–0.0016)

^b^ 0.0014 (0.0000–0.0043)

#### Trichuriasis

Trichuriasis demonstrated a weighted prevalence of 1.02% (95% CI: 0.15%-1.89%) nationally (**Tables 4** and **S4**). It was detected in 28 provinces, out of which six exceeded the average. The highest prevalence was found in Sichuan province (6.43%, 95% CI: 0.00%-16.75%), followed by Hainan (4.30%, 95% CI: 0.00%-10.25%) and Yunnan (4.18%, 95% CI: 1.37%-6.99%) (**Table 4** and **Fig 1D**). It was found in 32 of 46 ecozones, out of which eight exceed the average. The highest weighted prevalence was found in South Western Sichuan—Central Northern Yunnan Mountain ecozone (10.43%; 95% CI: 0.00%-23.87%), followed by Hainan Central Mountain ecozone (7.95%; 95% CI: 7.57%-8.33%) (**S4 Table** and **Fig 2D**). Trichuriasis were highly prevalent in the western regions, moderately so in central areas, but very much less common in the northern areas.

**Table 4:** Weighted prevalence and estimated population infected of trichuriasis by provinces in China in 2014–2015.

Province	No. sampled	No. infected	Prevalence (%)	Weighted prevalence (%) (95% CI)	Estimated population infected
**Beijing**	13401	4	0.03	0.02 (0.00–0.05)	401 (0–1325)
**Tianjin**	11107	0	0.00	0.00	0
**Hebei**	26526	0	0.00	0.00	0
**Shanxi**	19500	1	0.01	0.00 (0.00–0.01)	508 (0–1828)
**Neimenggu**	20132	5	0.02	0.08 (0.00–0.20)	8200 (0–20654)
**Liaoning**	26520	5	0.02	0.01 (0.00–0.03)	2043 (0–4912)
**Jilin**	19683	2	0.01	0.01 (0.00–0.04)	1675 (0–4836)
**Heilongjiang**	26456	1	0.00	0.00 (0.00–0.00)^a^	239 (0–713)
**Shanghai**	13547	2	0.01	0.01 (0.00–0.01)	125 (0–241)
**Jiangsu**	13275	2	0.02	0.01 (0.00–0.03)	3188 (0–9279)
**Zhejiang**	19935	20	0.10	0.05 (0.00–0.09)	9854 (0–18834)
**Anhui**	12300	23	0.19	0.41 (0.01–0.81)	126709 (3075–249065)
**Fujian**	7721	21	0.27	0.11 (0.02–0.20)	16362 (3014–30143)
**Jiangxi**	18649	152	0.82	1.11 (0.00–2.36)	273581 (0–580695)
**Shandong**	13620	79	0.58	0.52 (0.00–1.20)	248509 (0–576282)
**Henan**	26866	25	0.09	0.15 (0.01–0.30)	89485 (5782–173467)
**Hubei**	9215	27	0.29	0.25 (0.00–0.55)	67744 (0–149861)
**Hunan**	26389	39	0.15	0.15 (0.06–0.25)	54591 (21247–88529)
**Guangdong**	9309	42	0.45	0.25 (0.05–0.46)	80957 (16106–148178)
**Guangxi**	5702	84	1.47	1.88 (0.17–3.60)	496326 (44808–948879)
**Hainan**	2698	172	6.38	4.30 (0.00–10.25)	184085 (0–438943)
**Chongqing**	9250	21	0.23	0.28 (0.03–0.52)	33975 (3704–64200)
**Sichuan**	11403	360	3.16	6.43 (0.00–16.75)	3292254 (0–8575039)
**Guizhou**	7572	163	2.15	1.59 (0.78–2.40)	357555 (175363–539577)
**Yunnan**	5067	429	8.47	4.18 (1.37–6.99)	1242831 (407338–2078316)
**Xizang**	17939	57	0.32	0.29 (0.00–0.75)	6066 (0–15440)
**Shaanxi**	19900	5	0.03	0.00 (0.00–0.01)	543 (0–2045)
**Gansu**	17437	3	0.02	0.00 (0.00–0.01)	718 (0–1760)
**Qinghai**	12859	0	0.00	0.00	0
**Ningxia**	13346	7	0.05	0.05 (0.00–0.10)	1589 (0–3256)
**Xinjiang**	26886	5	0.02	0.02 (0.00–0.04)	2048 (0–4823)
**Total**	484210	1756	0.36	1.02 (0.15–1.89)	6602163 (972096–12248407)

^a^ 0.0015 (0.0000–0.0046)

### Age and gender distribution

**Fig 4:**
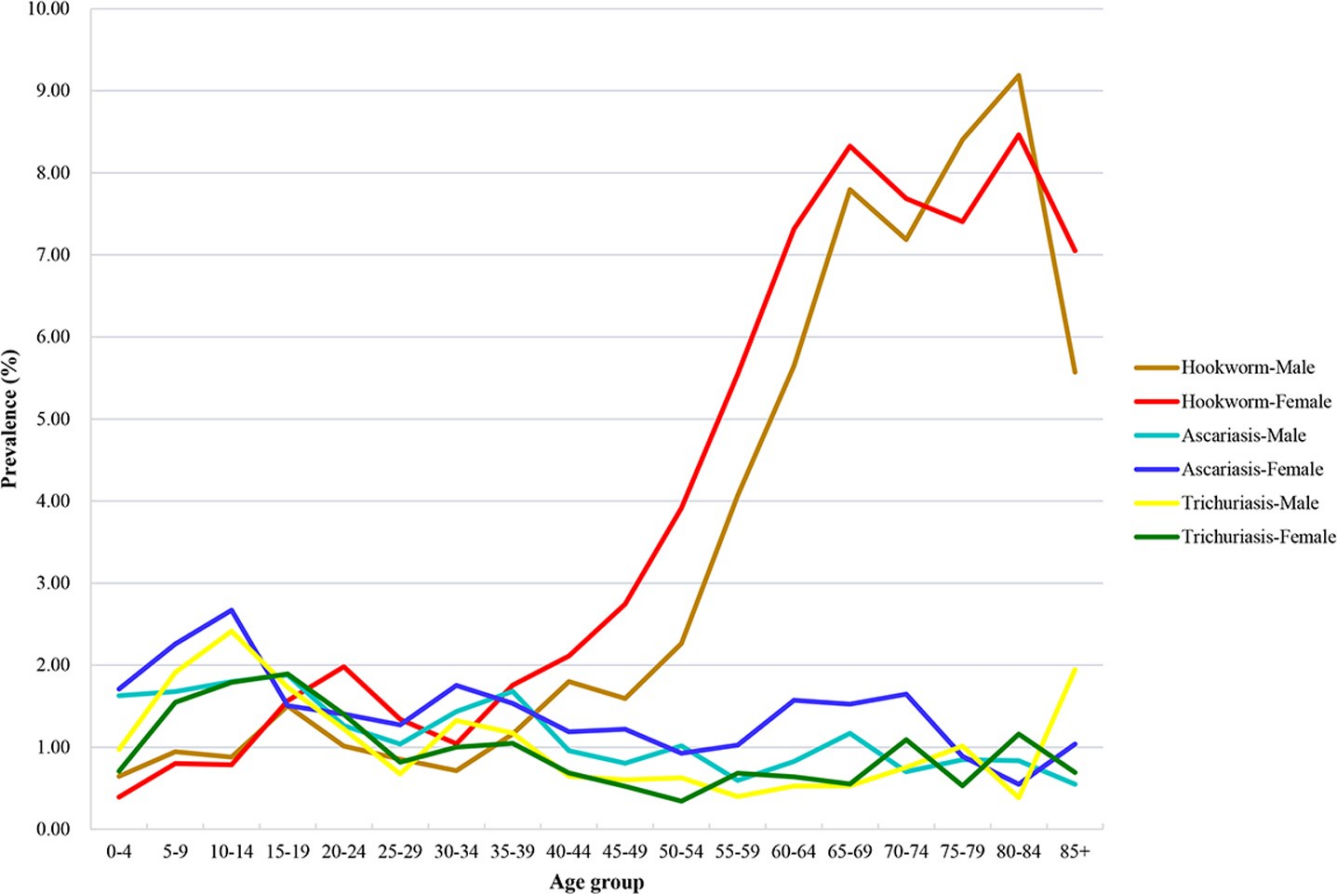
Weighted prevalence of soil-transmitted helminthiasis by ages and genders in China in 2014–2015.

Hookworm infections were highly prevalent in elderly, especially those over 60, who had a prevalence over 6% (**Fig 4** and **S5 Table**). The prevalence of hookworm infections was 2.29% (95% CI: 0.77%-3.81%) and 2.96% (95% CI: 0.93%-4.99%) in males and females, respectively. The difference in prevalence of ascariasis was not significant by age groups. However, a relatively high prevalence was demonstrated in those aged less than 20. The prevalence in age groups 0–4, 5–9, 10–14 and 15–19 was 1.66%, 1.94%, 2.20% and 1.70%, respectively. Additionally, the prevalence of ascariasis was 1.25% (95% CI: 0.46%-2.03%) and 1.48% (95% CI: 0.52%-2.44%) in males and females, respectively. Similarly, although the difference was not significant, the prevalence of trichuriasis was also a little high in those aged 5–19. The prevalence in age groups 5–9, 10–14 and 15–19 was 1.74%, 2.13% and 1.81%, respectively. The prevalence of trichuriasis was 1.06% (95% CI: 0.15%-1.97%) in males and 0.97% (95% CI: 0.14%-1.81%) in females.

### Estimated population infected

Overall, 29.12 million (95% CI: 15.88 million-42.32 million) people were estimated to be infected with at least one STH species and eight provinces harboured over 1 million infections; namely Sichuan (12.06 million), Yunnan (2.60 million), Guizhou (2.40 million), Guangdong (2.05 million), Guangxi (1.86 million), Hunan (1.63 million), Jiangxi (1.38 million) and Anhui (1.38 million).

16.97 million (95% CI: 5.57 million-28.39 million) people were estimated to have hookworm infections, among which 15.02 million (88.48%), 0.95 million (5.61%) and 1.00 million (5.91%) had light, moderate and heavy infections, respectively (**S6 Table**). Four provinces harboured over 1 million infections, namely Sichuan (7.45 million), Guangdong (1.76 million), Anhui (1.30 million) and Yunnan (1.14 million).

The number of people infected with *A*. *lumbricoides* was 8.83 million (95% CI: 3.18 million-14.45 million). Two provinces harboured over 1 million cases, namely Sichuan (3.50 million) and Guizhou (1.38 million). Among them, the numbers of light, moderate and heavy infections were 6.15 million (69.66%), 2.10 million (23.80%) and 0.58 million (6.54%), respectively (**S6 Table**).

About 6.60 million (95% CI: 0.97 million-12.25 million) people were estimated with *T*. *trichiura* infection, out of which 5.75 million (87.13%), 0.85 million (12.81%) and 0.04 million (0.05%) were light, moderate and heavy infections, respectively (**S6 Table**). Two provinces harboured over 1 million, namely Sichuan (3.29 million) and Yunnan (1.24 million).

## Discussion

This national survey updates the epidemiological map of STH in China. The prevalence of STH has decreased from 53.58% in 1988–1992 to 19.56% in 2001–2004 and then to 4.49% in this survey, while the number of estimated population with infection decreased from 646 million to 129 million and finally to 29 million [7,8]. It is thus obvious that significant control on STH has been achieved in rural China. Firstly, it is well established that endemicity of STH is related to poverty and inadequate access to health infrastructure [14,15], which have both improved over the almost 30 years since the first survey. The rate of poverty in rural China (defined as annual income below 2,300 Chinese Yuan) decreased from 73.5% of the population in 1990 to 5.7% in 2015 [16]. The coverage of piped water increased to 79.0% in 2014 [17], while the coverage of households with toilets in rural China increased to 78.4% in 2015, out of which 57.5% had non-hazardous toilets in which pathogens could be killed [17]. Secondly, huge intervention measures have been implemented with respect to control of STH. After the first national survey, such measures were applied in schools including health education, provision of safe water, improvement of sanitation and mass drug administration of needed pharmaceuticals [18]. After the second national survey, the measures in schools mentioned above have been applied in the community in general [19–21].

Although there was a significant decrease in STH infections, an imbalance of distribution is demonstrated due to multi-factorial impact. While the unsuitable natural environment of northern China (e.g. temperature, humidity) is a reason for the low endemicity of STH, especially hookworm infections, economic development has also been a crucial factor which has contributed to the decrease in STH infections [22]. As seen in the rural areas in eastern China, economic development impacts on STH in two ways. First, it reduces overall poverty leading to piped water and improved sanitation, but it also simultaneously promotes the implementation of health interventions. For example, the coverage of water supplement and sanitation is now usually over 90% in the eastern part of the country [17], while the economic development in western China is still lagging resulting in a lower coverage of water supplement and sanitation. Coverage has not yet reached 60% in the three most endemic provinces, namely Sichuan, Guizhou and Yunnan [17].

In this national survey, although the prevalences of ascariasis and trichuriasis were not significantly different by ages, but still a little higher in children. On the one hand, due to the poor hygiene, children are usually highly prevalent with both ascariasis and trichuriasis [4–6]. On the other hand, the overall low prevalence of ascariasis and trichuriasis weakened the difference. However, in western China, there still exist some factors contributing to the relatively high prevalence of ascariasis and trichuriasis in children, e.g. many siblings, low maternal education, low coverage of piped water and sanitation [23]. On the comparison, the prevalence of hookworm infections was significantly higher in adults. Human stool is still used as fertilizer in some remote areas in China and thus adults have more chance to be infected during agricultural activities [24].

Importantly, not only has the overall number of infections declined strongly, the predominance between the diseases has also changed, so that hookworm has changed from being the least common at 17% in 1988–1992 [7] to be the most common at 2.6% in 2014–2015. Ascariasis and trichuriasis are both more prevalent in children [4–6]. It is likely that school-based mass drug administration contributed to this large decrease in cases. Additionally, because of family planning, most families only raise one child, who therefore gets higher attention than of brothers and sister were around [23]. Furthermore, the provision of clean water and improvement in sanitation show higher effectiveness against ascariasis and trichuriasis than hookworm [14]. The composition of *Ancylostoma* spp., *N*. *americanus* and mix infections has changed from 43.17%, 42.20% and 14.63%, respectively, in 1988–1992 to 13.38%, 78.46% and 8.16% in this survey. The frequently used anthelmintic, albendazole, has a higher efficacy against *A*. *duodenale* (91.8%) than against *N*. *americanus* (75.0%) [25]. However, other factors might also contribute to this change, which deserve to be explored.

In this national survey, provinces, ecozones and economic level were included as the strata for the multiple-stage stratified cluster sampling, which increased the representativeness of sampled population. The estimation of sample size was based on the ecozones in each province, and thus this survey not only demonstrates the prevalence at national, provincial, ecozone level, but also at subprovincial (ecozones in each province) level, which would benefit the adoption of targeted strategy in different levels in future. However, there are several limitations in this survey. First, taking into consideration of the relatively low prevalence of STH in China, missed diagnoses cannot be avoided when only two smears from single sample were examined in this survey. Thus, the prevalence and estimated population under infection may be somewhat underestimated. Second, although *A*. *duodenale* and *N*. *americanus* are believed to be the predominant hookworm species in China, *A*. *ceylanicum* has also been reported in southern China [26]. Molecular techniques are needed in this situation, which deserves to be explored in further studies. After all, a One Health (veterinary and medical) approach to hookworm control is required in the areas where *A*. *ceylanicum* is found to be a common hookworm.

Overall, STH is now at a low endemic level in China. However, further control activities should be implemented in western parts of the country, especially in Sichuan, Yunnan and Guizhou provinces, which harbor over half of the STH cases discovered. Health education should be strengthened both in school and community. The predominance of hookworm infection requires implementation of community-based interventions. Additionally, water supply and improvement of sanitation should be prioritized in these areas. China has been listed among the countries most feasible to achieve the transmission interruption of STH [22,27]. Significantly, the interruption of STH should firstly be attempted in the northern and eastern China, where the prevalence has decreased to less than 1%. China has eliminated poverty in 2020 and established what is called healthy China by 2030, a plan in which the elimination of the STH could be integrated [28].

## Supporting information

S1 STROBE Checklist STROBE (Strengthening The Reporting of OBservational Studies in Epidemiology) Checklist. (PDF)

S1 Table Weighted prevalence and estimated population infected of soil-transmitted helminthiasis by ecozones in China in 2014–2015. (DOCX)

S2 Table Weighted prevalence and estimated population infected of hookworm by ecozones in China in 2014–2015. (DOCX)

S3 Table Weighted prevalence and estimated population infected of ascariasis by ecozones in China in 2014–2015. (DOCX)

S4 Table Weighted prevalence and estimated population infected of trichuriasis by ecozones in China in 2014–2015. (DOCX)

S5 Table Weighted prevalence of soil-transmitted helminthiasis by ages and genders in China in 2014–2015. (DOCX)

S6 Table Estimated population infected of soil-transmitted helminthiasis by infection intensity and provinces in China in 2014–2015. (DOCX)
