# Supplementary material for: Soil-transmitted helminthiasis in China: A national survey in 2014-2015
Source: PLoS Negl Trop Dis. 2021 Oct 19;15(10):e0009710. doi: 10.1371/journal.pntd.0009710 (PMC8555824; doi:10.1371/journal.pntd.0009710)
Supplement: S1 Table — (DOCX) [file pntd.0009710.s002.docx]

**S1 Table.** Weighted prevalence and estimated population infected of soil-transmitted helminthiasis by ecozones in China in 2014-2015

| **Ecozone** | **No. sampled** | **No. infected** | **Prevalence (%)** | **Weighted prevalence (%) (95% CI)** | **Estimated population infected** |
| --- | --- | --- | --- | --- | --- |
| **I-02** | 6283 | 0 | 0.00 | 0.00 | 0 |
| **I-03** | 6574 | 0 | 0.00 | 0.00 | 0 |
| **I-04** | 19997 | 74 | 0.37 | 0.48 (0.05-0.90) | 62456 (6531-117551) |
| **I-05** | 19828 | 3 | 0.02 | 0.00 (0.00-0.01) | 374 (0-1340) |
| **I-06** | 6653 | 14 | 0.21 | 0.20 (0.00-0.42) | 5154 (0-11015) |
| **I-07** | 6825 | 16 | 0.23 | 0.29 (0.00-0.62) | 11489 (0-24932) |
| **I-08** | 13397 | 127 | 0.95 | 0.89 (0.00-1.93) | 291276 (0-632772) |
| **I-09** | 19976 | 5 | 0.03 | 0.03 (0.00-0.10) | 2360 (0-6930) |
| **I-10** | 37965 | 64 | 0.17 | 0.07 (0.01-0.14) | 24355 (3299-46181) |
| **I-11** | 13083 | 150 | 1.15 | 2.51 (0.01-5.00) | 247633 (988-493773) |
| **I-12** | 24151 | 271 | 1.12 | 0.93 (0.18-1.68) | 204586 (39445-368156) |
| **I-13** | 29809 | 178 | 0.60 | 1.30 (0.00-2.94) | 1549871 (0-3502965) |
| **I-14** | 9743 | 115 | 1.18 | 1.05 (0.00-2.39) | 116088 (0-263917) |
| **I-15** | 24642 | 502 | 2.04 | 1.31 (0.39-2.23) | 476705 (142376-814101) |
| **I-16** | 13323 | 81 | 0.61 | 0.31 (0.16-0.47) | 48926 (24942-73268) |
| **I-17** | 16875 | 707 | 4.19 | 2.95 (0.69-5.21) | 805496 (188295-1421766) |
| **I-18** | 2380 | 196 | 8.24 | 8.13 (6.94-9.32) | 402178 (343389-461150) |
| **I-19** | 7388 | 1211 | 16.39 | 22.16 (10.84-33.48) | 8055880 (3941222-12172703) |
| **I-20** | 9644 | 240 | 2.49 | 2.30 (0.82-3.78) | 99626 (35517-163726) |
| **I-21** | 15143 | 559 | 3.69 | 1.88 (0.96-2.79) | 531292 (271707-789649) |
| **I-22** | 13426 | 617 | 4.60 | 4.01 (2.88-5.13) | 912572 (656012-1168521) |
| **I-23** | 12618 | 988 | 7.83 | 9.27 (5.87-12.66) | 2146712 (1359723-2932554) |
| **I-24** | 1504 | 115 | 7.65 | 6.15 (4.74-7.55) | 647272 (499039-794883) |
| **I-25** | 5014 | 827 | 16.49 | 15.88 (0.00-33.23) | 5508666 (0-11528969) |
| **I-26** | 15455 | 401 | 2.59 | 3.14 (1.54-4.73) | 716700 (352024-1081216) |
| **I-28** | 9513 | 655 | 6.89 | 6.71 (2.70-10.73) | 3016298 (1213642-4823103) |
| **I-29** | 2370 | 2 | 0.08 | 0.14 (0.04-0.24) | 1150 (336-2014) |
| **I-31** | 5612 | 565 | 10.07 | 11.83 (6.51-17.15) | 1996928 (1098810-2894715) |
| **I-32** | 1381 | 142 | 10.28 | 11.06 (9.76-12.36) | 422688 (373012-472380) |
| **I-33** | 1317 | 248 | 18.83 | 21.92 (17.52-26.33) | 100968 (80685-121257) |
| **I-34** | 6999 | 1 | 0.01 | 0.00 (0.00-0.01) | 74 (0-185) |
| **I-35** | 6548 | 12 | 0.18 | 0.17 (0.17-0.17) | 951 (951-951) |
| **II-01** | 12979 | 8 | 0.06 | 0.16 (0.00-0.35) | 8642 (0-19343) |
| **II-02** | 6627 | 94 | 1.42 | 1.28 (0.58-1.98) | 22469 (10185-34770) |
| **II-03** | 4313 | 76 | 1.76 | 2.41 (0.00-4.97) | 53071 (0-109352) |
| **II-04** | 11349 | 117 | 1.03 | 0.34 (0.00-0.72) | 8137 (0-17232) |
| **II-05** | 6798 | 81 | 1.19 | 1.25 (0.55-1.96) | 14656 (6437-22937) |
| **II-06** | 6772 | 61 | 0.90 | 0.69 (0.03-1.35) | 14154 (618-27813) |
| **II-07** | 6808 | 161 | 2.36 | 1.15 (0.00-2.97) | 21218 (0-54622) |
| **II-08** | 6508 | 8 | 0.12 | 0.17 (0.00-0.43) | 11586 (0-30043) |
| **III-01** | 4292 | 65 | 1.51 | 1.45 (0.94-1.97) | 21541 (13952-29239) |
| **III-02** | 4265 | 53 | 1.24 | 1.17 (0.85-1.50) | 2700 (1956-3452) |
| **III-04** | 6580 | 40 | 0.61 | 0.43 (0.09-0.78) | 18444 (3841-33286) |
| **III-05** | 4371 | 25 | 0.57 | 0.30 (0.00-0.61) | 334 (0-688) |
| **III-07** | 10433 | 727 | 6.97 | 7.99 (5.13-10.84) | 510002 (327638-692319) |
| **III-08** | 6679 | 79 | 1.18 | 0.46 (0.00-1.34) | 4330 (0-12680) |
| **Total** | 484210 | 10681 | 2.21 | 4.49 (2.45-6.53) | 29118009 (15877565-42318571) |
