# Supplementary material for: Soil-transmitted helminthiasis in China: A national survey in 2014-2015
Source: PLoS Negl Trop Dis. 2021 Oct 19;15(10):e0009710. doi: 10.1371/journal.pntd.0009710 (PMC8555824; doi:10.1371/journal.pntd.0009710)
Supplement: S2 Table — (DOCX) [file pntd.0009710.s003.docx]

**S2 Table.** Weighted prevalence and estimated population infected of hookworm by ecozones in China in 2014-2015

| **Ecozone** | **No. sampled** | **No. infected** | **Prevalence (%)** | **Weighted prevalence (%) (95% CI)** | **Estimated population infected** | | | |
| --- | --- | --- | --- | --- | --- | --- | --- | --- |
|  |  |  |  |  | **Totally (95% CI)** | **Light** | **Moderate** | **Heavy** |
| **I-02** | 6283 | 0 | 0.00 | 0.00 | 0 | 0 | 0 | 0 |
| **I-03** | 6574 | 0 | 0.00 | 0.00 | 0 | 0 | 0 | 0 |
| **I-04** | 19997 | 0 | 0.00 | 0.00 | 0 | 0 | 0 | 0 |
| **I-05** | 19828 | 0 | 0.00 | 0.00 | 0 | 0 | 0 | 0 |
| **I-06** | 6653 | 0 | 0.00 | 0.00 | 0 | 0 | 0 | 0 |
| **I-07** | 6825 | 0 | 0.00 | 0.00 | 0 | 0 | 0 | 0 |
| **I-08** | 13397 | 0 | 0.00 | 0.00 | 0 | 0 | 0 | 0 |
| **I-09** | 19976 | 0 | 0.00 | 0.00 | 0 | 0 | 0 | 0 |
| **I-10** | 37965 | 3 | 0.01 | 0.00 (0.00-0.00)^a^ | 330 (0-825) | 330 | 0 | 0 |
| **I-11** | 13083 | 0 | 0.00 | 0.00 | 0 | 0 | 0 | 0 |
| **I-12** | 24151 | 3 | 0.01 | 0.00 (0.00-0.01) | 735 (0-2191) | 735 | 0 | 0 |
| **I-13** | 29809 | 122 | 0.41 | 1.01 (0.00-2.61) | 1206599 (0-3109775) | 1140557 | 39988 | 26054 |
| **I-14** | 9743 | 84 | 0.86 | 0.91 (0.00-2.12) | 100524 (0-234102) | 95235 | 4296 | 993 |
| **I-15** | 24642 | 157 | 0.64 | 0.51 (0.00-1.26) | 185874 (0-459986) | 171546 | 6762 | 7566 |
| **I-16** | 13323 | 56 | 0.42 | 0.16 (0.05-0.28) | 25473 (7794-43649) | 25115 | 95 | 263 |
| **I-17** | 16875 | 372 | 2.20 | 1.06 (0.09-2.04) | 290547 (24560-556699) | 262552 | 11466 | 16529 |
| **I-18** | 2380 | 135 | 5.67 | 4.95 (3.73-6.18) | 245027 (184559-305784) | 218782 | 15095 | 11149 |
| **I-19** | 7388 | 914 | 12.37 | 20.34 (8.27-32.41) | 7394051 (3006818-11783671) | 5989224 | 644349 | 760478 |
| **I-20** | 9644 | 224 | 2.32 | 2.08 (0.61-3.56) | 90211 (26421-154197) | 83277 | 2481 | 4454 |
| **I-21** | 15143 | 523 | 3.45 | 1.72 (0.88-2.55) | 485457 (249065-721722) | 441207 | 25033 | 19217 |
| **I-22** | 13426 | 460 | 3.43 | 2.87 (1.76-3.98) | 654217 (400896-906572) | 606965 | 24600 | 22652 |
| **I-23** | 12618 | 700 | 5.55 | 6.32 (3.86-8.78) | 1463723 (894128-2033793) | 1350131 | 51390 | 62202 |
| **I-24** | 1504 | 2 | 0.13 | 0.10 (0.00-0.27) | 10998 (0-28426) | 10998 | 0 | 0 |
| **I-25** | 5014 | 176 | 3.51 | 1.45 (0.35-2.55) | 502755 (121431-884709) | 479795 | 4704 | 18257 |
| **I-26** | 15455 | 233 | 1.51 | 1.71 (0.54-2.88) | 389851 (123437-658330) | 370167 | 8307 | 11377 |
| **I-28** | 9513 | 454 | 4.77 | 5.07 (1.23-8.90) | 2276811 (552881-4000523) | 2181886 | 82754 | 12171 |
| **I-29** | 2370 | 0 | 0.00 | 0.00 | 0 | 0 | 0 | 0 |
| **I-31** | 5612 | 376 | 6.70 | 6.61 (2.95-10.26) | 1115158 (497925-1731765) | 1077476 | 17185 | 20498 |
| **I-32** | 1381 | 86 | 6.23 | 7.20 (2.17-12.23) | 275209 (82934-467411) | 263812 | 2928 | 8468 |
| **I-33** | 1317 | 139 | 10.55 | 15.54 (9.97-21.10) | 71560 (45915-97171) | 70579 | 683 | 298 |
| **I-34** | 6999 | 0 | 0.00 | 0.00 | 0 | 0 | 0 | 0 |
| **I-35** | 6548 | 0 | 0.00 | 0.00 | 0 | 0 | 0 | 0 |
| **II-01** | 12979 | 0 | 0.00 | 0.00 | 0 | 0 | 0 | 0 |
| **II-02** | 6627 | 5 | 0.08 | 0.11 (0.00-0.29) | 1904 (0-5093) | 1904 | 0 | 0 |
| **II-03** | 4313 | 0 | 0.00 | 0.00 | 0 | 0 | 0 | 0 |
| **II-04** | 11349 | 0 | 0.00 | 0.00 | 0 | 0 | 0 | 0 |
| **II-05** | 6798 | 0 | 0.00 | 0.00 | 0 | 0 | 0 | 0 |
| **II-06** | 6772 | 0 | 0.00 | 0.00 | 0 | 0 | 0 | 0 |
| **II-07** | 6808 | 0 | 0.00 | 0.00 | 0 | 0 | 0 | 0 |
| **II-08** | 6508 | 0 | 0.00 | 0.00 | 0 | 0 | 0 | 0 |
| **III-01** | 4292 | 0 | 0.00 | 0.00 | 0 | 0 | 0 | 0 |
| **III-02** | 4265 | 0 | 0.00 | 0.00 | 0 | 0 | 0 | 0 |
| **III-04** | 6580 | 0 | 0.00 | 0.00 | 0 | 0 | 0 | 0 |
| **III-05** | 4371 | 0 | 0.00 | 0.00 | 0 | 0 | 0 | 0 |
| **III-07** | 10433 | 199 | 1.91 | 2.94 (0.00-6.35) | 187508 (0-405556) | 177148 | 10211 | 149 |
| **III-08** | 6679 | 0 | 0.00 | 0.00 | 0 | 0 | 0 | 0 |
| **Total** | 484210 | 5423 | 1.12 | 2.62 (0.86-4.38) | 16974524 (5573349-28385198) | 15019422 | 952326 | 1002776 |

^a^ 0.0010 (0.0000-0.0025)
