# Supplementary material for: Soil-transmitted helminthiasis in China: A national survey in 2014-2015
Source: PLoS Negl Trop Dis. 2021 Oct 19;15(10):e0009710. doi: 10.1371/journal.pntd.0009710 (PMC8555824; doi:10.1371/journal.pntd.0009710)
Supplement: S3 Table — (DOCX) [file pntd.0009710.s004.docx]

**S3 Table.** Weighted prevalence and estimated population infected of ascariasis by ecozones in China in 2014-2015

| **Ecozone** | **No. sampled** | **No. infected** | **Prevalence (%)** | **Weighted prevalence (%) (95% CI)** | **Estimated population infected** | | | |
| --- | --- | --- | --- | --- | --- | --- | --- | --- |
|  |  |  |  |  | **Totally (95% CI)** | **Light** | **Moderate** | **Heavy** |
| **I-02** | 6283 | 0 | 0.00 | 0.00 | 0 | 0 | 0 | 0 |
| **I-03** | 6574 | 0 | 0.00 | 0.00 | 0 | 0 | 0 | 0 |
| **I-04** | 19997 | 71 | 0.36 | 0.46 (0.04-0.89) | 60542 (5224-116245) | 48283 | 12259 | 0 |
| **I-05** | 19828 | 3 | 0.02 | 0.00 (0.00-0.01) | 374 (0-1340) | 374 | 0 | 0 |
| **I-06** | 6653 | 14 | 0.21 | 0.20 (0.00-0.42) | 5154 (0-11015) | 4040 | 1114 | 0 |
| **I-07** | 6825 | 15 | 0.22 | 0.28 (0.00-0.60) | 11067 (0-24127) | 9986 | 1081 | 0 |
| **I-08** | 13397 | 52 | 0.39 | 0.16 (0.00-0.33) | 52720 (0-108194) | 47869 | 2908 | 1944 |
| **I-09** | 19976 | 5 | 0.03 | 0.03 (0.00-0.10) | 2360 (0-6930) | 2360 | 0 | 0 |
| **I-10** | 37965 | 52 | 0.14 | 0.07 (0.00-0.13) | 22156 (0-42883) | 20640 | 1244 | 272 |
| **I-11** | 13083 | 150 | 1.15 | 2.51 (0.01-5.00) | 247633 (988-493773) | 170473 | 45599 | 31561 |
| **I-12** | 24151 | 264 | 1.09 | 0.93 (0.18-1.68) | 203440 (39445-368156) | 200802 | 2419 | 219 |
| **I-13** | 29809 | 40 | 0.13 | 0.17 (0.01-0.33) | 202783 (11915-393190) | 201216 | 1568 | 0 |
| **I-14** | 9743 | 23 | 0.24 | 0.09 (0.00-0.19) | 9504 (0-20981) | 9330 | 173 | 0 |
| **I-15** | 24642 | 329 | 1.34 | 0.75 (0.32-1.18) | 273677 (116822-430780) | 251188 | 21716 | 773 |
| **I-16** | 13323 | 16 | 0.12 | 0.14 (0.09-0.18) | 21244 (14030-28060) | 21244 | 0 | 0 |
| **I-17** | 16875 | 214 | 1.27 | 1.01 (0.00-2.11) | 275196 (0-575802) | 243468 | 20376 | 11352 |
| **I-18** | 2380 | 68 | 2.86 | 3.41 (0.95-5.88) | 168897 (47006-290940) | 144782 | 24114 | 0 |
| **I-19** | 7388 | 309 | 4.18 | 1.39 (0.59-2.18) | 503570 (214513-792607) | 423340 | 78046 | 2184 |
| **I-20** | 9644 | 6 | 0.06 | 0.07 (0.00-0.14) | 3046 (0-6064) | 1450 | 1596 | 0 |
| **I-21** | 15143 | 10 | 0.07 | 0.04 (0.01-0.07) | 11166 (2830-19812) | 10443 | 722 | 0 |
| **I-22** | 13426 | 132 | 0.98 | 0.97 (0.49-1.45) | 220212 (111613-330284) | 196850 | 23362 | 0 |
| **I-23** | 12618 | 285 | 2.26 | 2.97 (1.23-4.71) | 688848 (284916-1091021) | 475626 | 185465 | 27757 |
| **I-24** | 1504 | 100 | 6.65 | 5.30 (3.68-6.93) | 558477 (387440-729608) | 389911 | 162315 | 6251 |
| **I-25** | 5014 | 487 | 9.71 | 10.02 (0.00-24.57) | 3476630 (0-8524429) | 1916496 | 1099910 | 460224 |
| **I-26** | 15455 | 138 | 0.89 | 1.03 (0.26-1.79) | 234362 (59433-409171) | 187148 | 47214 | 0 |
| **I-28** | 9513 | 157 | 1.65 | 1.18 (0.51-1.85) | 531020 (229243-831569) | 395306 | 107852 | 27863 |
| **I-29** | 2370 | 0 | 0.00 | 0.00 | 0 | 0 | 0 | 0 |
| **I-31** | 5612 | 131 | 2.33 | 3.58 (2.14-5.02) | 604679 (361206-847316) | 412341 | 186413 | 5925 |
| **I-32** | 1381 | 0 | 0.00 | 0.00 | 0 | 0 | 0 | 0 |
| **I-33** | 1317 | 2 | 0.15 | 0.20 (0.13-0.27) | 933 (599-1243) | 882 | 50 | 0 |
| **I-34** | 6999 | 1 | 0.01 | 0.00 (0.00-0.01) | 74 (0-185) | 74 | 0 | 0 |
| **I-35** | 6548 | 10 | 0.15 | 0.15 (0.15-0.15) | 826 (826-826) | 716 | 110 | 0 |
| **II-01** | 12979 | 5 | 0.04 | 0.02 (0.00-0.04) | 975 (0-2211) | 975 | 0 | 0 |
| **II-02** | 6627 | 86 | 1.30 | 1.10 (0.41-1.80) | 19388 (7200-31609) | 19229 | 159 | 0 |
| **II-03** | 4313 | 76 | 1.76 | 2.41 (0.00-4.97) | 53071 (0-109352) | 53071 | 0 | 0 |
| **II-04** | 11349 | 115 | 1.01 | 0.33 (0.00-0.72) | 8005 (0-17232) | 8005 | 0 | 0 |
| **II-05** | 6798 | 81 | 1.19 | 1.25 (0.55-1.96) | 14656 (6437-22937) | 14656 | 0 | 0 |
| **II-06** | 6772 | 56 | 0.83 | 0.59 (0.00-1.27) | 12106 (0-26165) | 12106 | 0 | 0 |
| **II-07** | 6808 | 161 | 2.36 | 1.15 (0.00-2.97) | 21218 (0-54622) | 21148 | 71 | 0 |
| **II-08** | 6508 | 8 | 0.12 | 0.17 (0.00-0.43) | 11586 (0-30043) | 11586 | 0 | 0 |
| **III-01** | 4292 | 65 | 1.51 | 1.45 (0.94-1.97) | 21541 (13952-29239) | 18996 | 2545 | 0 |
| **III-02** | 4265 | 53 | 1.24 | 1.17 (0.85-1.50) | 2700 (1956-3452) | 2444 | 256 | 0 |
| **III-04** | 6580 | 38 | 0.58 | 0.37 (0.01-0.72) | 15578 (427-30725) | 11759 | 3600 | 219 |
| **III-05** | 4371 | 25 | 0.57 | 0.30 (0.00-0.61) | 334 (0-688) | 334 | 0 | 0 |
| **III-07** | 10433 | 413 | 3.96 | 3.92 (1.84-5.99) | 250236 (117515-382564) | 182807 | 66620 | 808 |
| **III-08** | 6679 | 77 | 1.15 | 0.44 (0.00-1.32) | 4192 (0-12491) | 4132 | 60 | 0 |
| **Total** | 484210 | 4343 | 0.90 | 1.36 (0.49-2.23) | 8826171 (3175513-14451824) | 6147884 | 2100937 | 577351 |
