# Supplementary material for: Soil-transmitted helminthiasis in China: A national survey in 2014-2015
Source: PLoS Negl Trop Dis. 2021 Oct 19;15(10):e0009710. doi: 10.1371/journal.pntd.0009710 (PMC8555824; doi:10.1371/journal.pntd.0009710)
Supplement: S4 Table — (DOCX) [file pntd.0009710.s005.docx]

**S4 Table.** Weighted prevalence and estimated population infected of trichuriasis by ecozones in China in 2014-2015

| **Ecozone** | **No. sampled** | **No. infected** | **Prevalence (%)** | **Weighted prevalence (%) (95% CI)** | **Estimated population infected** | | | |
| --- | --- | --- | --- | --- | --- | --- | --- | --- |
|  |  |  |  |  | **Totally (95% CI)** | **Light** | **Moderate** | **Heavy** |
| **I-02** | 6283 | 0 | 0.00 | 0.00 | 0 | 0 | 0 | 0 |
| **I-03** | 6574 | 0 | 0.00 | 0.00 | 0 | 0 | 0 | 0 |
| **I-04** | 19997 | 3 | 0.02 | 0.01 (0.00-0.04) | 1914 (0-5224) | 1914 | 0 | 0 |
| **I-05** | 19828 | 0 | 0.00 | 0.00 | 0 | 0 | 0 | 0 |
| **I-06** | 6653 | 0 | 0.00 | 0.00 | 0 | 0 | 0 | 0 |
| **I-07** | 6825 | 1 | 0.01 | 0.01 (0.00-0.03) | 422 (0-1206) | 422 | 0 | 0 |
| **I-08** | 13397 | 82 | 0.61 | 0.76 (0.00-1.74) | 248319 (0-570479) | 223876 | 22671 | 1771 |
| **I-09** | 19976 | 0 | 0.00 | 0.00 | 0 | 0 | 0 | 0 |
| **I-10** | 37965 | 13 | 0.03 | 0.01 (0.00-0.01) | 2270 (0-3299) | 2160 | 110 | 0 |
| **I-11** | 13083 | 0 | 0.00 | 0.00 | 0 | 0 | 0 | 0 |
| **I-12** | 24151 | 5 | 0.02 | 0.00 (0.00-0.01) | 791 (0-2191) | 791 | 0 | 0 |
| **I-13** | 29809 | 18 | 0.06 | 0.17 (0.00-0.34) | 202034 (0-405105) | 202034 | 0 | 0 |
| **I-14** | 9743 | 12 | 0.12 | 0.08 (0.02-0.13) | 8309 (2209-14355) | 7063 | 1245 | 0 |
| **I-15** | 24642 | 26 | 0.11 | 0.06 (0.01-0.11) | 20526 (3651-40157) | 19927 | 599 | 0 |
| **I-16** | 13323 | 11 | 0.08 | 0.02 (0.00-0.04) | 2580 (0-6236) | 2580 | 0 | 0 |
| **I-17** | 16875 | 144 | 0.85 | 1.06 (0.00-2.29) | 290344 (0-624922) | 283940 | 6404 | 0 |
| **I-18** | 2380 | 11 | 0.46 | 0.51 (0.11-0.92) | 25412 (5443-45521) | 25412 | 0 | 0 |
| **I-19** | 7388 | 72 | 0.97 | 1.11 (0.78-1.43) | 402057 (283593-519921) | 402057 | 0 | 0 |
| **I-20** | 9644 | 12 | 0.12 | 0.18 (0.05-0.30) | 7608 (2166-12994) | 6375 | 1233 | 0 |
| **I-21** | 15143 | 30 | 0.20 | 0.13 (0.03-0.24) | 38007 (8491-67927) | 36578 | 1429 | 0 |
| **I-22** | 13426 | 37 | 0.28 | 0.23 (0.10-0.37) | 52997 (22778-84279) | 47862 | 5135 | 0 |
| **I-23** | 12618 | 47 | 0.37 | 0.57 (0.00-1.22) | 132934 (0-282600) | 132934 | 0 | 0 |
| **I-24** | 1504 | 23 | 1.53 | 1.34 (0.00-2.83) | 140909 (0-297950) | 140909 | 0 | 0 |
| **I-25** | 5014 | 389 | 7.76 | 10.43 (0.00-23.87) | 3618439 (0-8281568) | 2950883 | 667556 | 0 |
| **I-26** | 15455 | 35 | 0.23 | 0.43 (0.00-0.92) | 97591 (0-210300) | 83385 | 14206 | 0 |
| **I-28** | 9513 | 109 | 1.15 | 0.79 (0.00-1.93) | 356163 (0-867529) | 355695 | 468 | 0 |
| **I-29** | 2370 | 2 | 0.08 | 0.14 (0.04-0.24) | 1150 (336-2014) | 1150 | 0 | 0 |
| **I-31** | 5612 | 108 | 1.92 | 3.73 (0.49-6.96) | 629116 (82706-1174765) | 619556 | 9560 | 0 |
| **I-32** | 1381 | 56 | 4.06 | 3.86 (0.00-10.19) | 147479 (0-389446) | 43968 | 102453 | 1059 |
| **I-33** | 1317 | 116 | 8.81 | 7.95 (7.57-8.33) | 36606 (34862-38362) | 28608 | 7543 | 455 |
| **I-34** | 6999 | 0 | 0.00 | 0.00 | 0 | 0 | 0 | 0 |
| **I-35** | 6548 | 2 | 0.03 | 0.02 (0.02-0.02) | 125 (125-125) | 125 | 0 | 0 |
| **II-01** | 12979 | 3 | 0.02 | 0.14 (0.00-0.34) | 7667 (0-18791) | 7667 | 0 | 0 |
| **II-02** | 6627 | 3 | 0.05 | 0.07 (0.00-0.16) | 1177 (0-2810) | 1177 | 0 | 0 |
| **II-03** | 4313 | 0 | 0.00 | 0.00 | 0 | 0 | 0 | 0 |
| **II-04** | 11349 | 3 | 0.03 | 0.01 (0.00-0.03) | 323 (0-718) | 212 | 111 | 0 |
| **II-05** | 6798 | 0 | 0.00 | 0.00 | 0 | 0 | 0 | 0 |
| **II-06** | 6772 | 5 | 0.07 | 0.10 (0.06-0.14) | 2048 (1236-2884) | 2048 | 0 | 0 |
| **II-07** | 6808 | 0 | 0.00 | 0.00 | 0 | 0 | 0 | 0 |
| **II-08** | 6508 | 0 | 0.00 | 0.00 | 0 | 0 | 0 | 0 |
| **III-01** | 4292 | 0 | 0.00 | 0.00 | 0 | 0 | 0 | 0 |
| **III-02** | 4265 | 0 | 0.00 | 0.00 | 0 | 0 | 0 | 0 |
| **III-04** | 6580 | 2 | 0.03 | 0.07 (0.00-0.13) | 2866 (0-5548) | 2866 | 0 | 0 |
| **III-05** | 4371 | 0 | 0.00 | 0.00 | 0 | 0 | 0 | 0 |
| **III-07** | 10433 | 374 | 3.58 | 1.94 (0.74-3.14) | 123840 (47262-200543) | 118380 | 5153 | 307 |
| **III-08** | 6679 | 2 | 0.03 | 0.01 (0.00-0.04) | 138 (0-379) | 138 | 0 | 0 |
| **Total** | 484210 | 1756 | 0.36 | 1.02 (0.15-1.89) | 6602163 (972096-12248407) | 5752696 | 845876 | 3592 |
