# Supplementary material for: Soil-transmitted helminthiasis in China: A national survey in 2014-2015
Source: PLoS Negl Trop Dis. 2021 Oct 19;15(10):e0009710. doi: 10.1371/journal.pntd.0009710 (PMC8555824; doi:10.1371/journal.pntd.0009710)
Supplement: S5 Table — (DOCX) [file pntd.0009710.s006.docx]

**S5 Table.** Weighted prevalence of soil-transmitted helminthiasis by ages and genders in China in 2014-2015

| **Age groups** | **Hookworm in male (%)** | **Hookworm in female (%)** | **Ascariasis in male (%)** | **Ascariasis in female (%)** | **Trichuriasis in male (%)** | **Trichuriasis in female (%)** |
| --- | --- | --- | --- | --- | --- | --- |
| **0-4** | 0.65 (0.17-1.12) | 0.39 (0.03-0.76) | 1.63 (0.59-2.67) | 1.71 (0.24-3.18) | 0.97 (0.00-2.04) | 0.71 (0.00-1.56) |
| **5-9** | 0.94 (0.08-1.81) | 0.80 (0.35-1.25) | 1.68 (0.85-2.50) | 2.26 (0.66-3.86) | 1.91 (0.57-3.25) | 1.55 (0.17-2.92) |
| **10-14** | 0.88 (0.23-1.53) | 0.78 (0.25-1.32) | 1.80 (0.52-3.09) | 2.67 (0.51-4.83) | 2.41 (0.28-4.54) | 1.79 (0.00-3.69) |
| **15-19** | 1.50 (0.22-2.79) | 1.56 (0.56-2.56) | 1.88 (0.40-3.36) | 1.51 (0.09-2.92) | 1.73 (0.00-3.91) | 1.89 (0.13-3.65) |
| **20-24** | 1.01 (0.22-1.80) | 1.98 (0.64-3.32) | 1.26 (0.11-2.42) | 1.40 (0.00-3.08) | 1.22 (0.00-2.46) | 1.40 (0.00-3.05) |
| **25-29** | 0.85 (0.42-1.29) | 1.34 (0.58-2.10) | 1.04 (0.32-1.76) | 1.27 (0.58-1.96) | 0.67 (0.04-1.31) | 0.82 (0.05-1.59) |
| **30-34** | 0.71 (0.12-1.31) | 1.04 (0.58-1.51) | 1.43 (0.08-2.79) | 1.75 (0.00-3.86) | 1.33 (0.00-2.82) | 1.00 (0.00-2.28) |
| **35-39** | 1.16 (0.45-1.86) | 1.76 (0.60-2.92) | 1.68 (0.00-3.68) | 1.53 (0.29-2.78) | 1.17 (0.00-2.76) | 1.05 (0.00-2.13) |
| **40-44** | 1.80 (0.87-2.73) | 2.11 (1.04-3.18) | 0.96 (0.23-1.68) | 1.19 (0.33-2.05) | 0.65 (0.00-1.45) | 0.69 (0.00-1.46) |
| **45-49** | 1.59 (1.01-2.18) | 2.75 (1.11-4.38) | 0.81 (0.49-1.12) | 1.22 (0.70-1.74) | 0.60 (0.05-1.15) | 0.52 (0.08-0.97) |
| **50-54** | 2.26 (1.09-3.43) | 3.91 (1.40-6.43) | 1.02 (0.33-1.71) | 0.93 (0.58-1.27) | 0.63 (0.14-1.11) | 0.34 (0.13-0.56) |
| **55-59** | 4.07 (0.98-7.15) | 5.55 (1.11-9.98) | 0.59 (0.37-0.81) | 1.03 (0.63-1.42) | 0.40 (0.11-0.69) | 0.68 (0.17-1.19) |
| **60-64** | 5.65 (0.26-11.03) | 7.31 (0.71-13.91) | 0.83 (0.51-1.14) | 1.57 (0.99-2.15) | 0.53 (0.22-0.83) | 0.64 (0.28-1.00) |
| **65-69** | 7.80 (0.80-14.80) | 8.33 (1.46-15.19) | 1.17 (0.71-1.63) | 1.52 (0.88-2.17) | 0.53 (0.21-0.85) | 0.55 (0.25-0.85) |
| **70-74** | 7.19 (0.51-13.87) | 7.69 (2.38-12.99) | 0.70 (0.35-1.06) | 1.65 (0.98-2.32) | 0.75 (0.14-1.37) | 1.09 (0.00-2.25) |
| **75-79** | 8.40 (1.00-15.81) | 7.40 (0.26-14.55) | 0.85 (0.43-1.27) | 0.89 (0.40-1.38) | 1.01 (0.38-1.65) | 0.53 (0.05-1.01) |
| **80-84** | 9.19 (0.00-18.38) | 8.46 (1.94-14.99) | 0.84 (0.04-1.63) | 0.55 (0.19-0.90) | 0.38 (0.06-0.70) | 1.16 (0.01-2.31) |
| **85+** | 5.57 (0.05-11.10) | 7.05 (1.57-12.53) | 0.55 (0.00-1.12) | 1.04 (0.00-2.07) | 1.95 (0.00-5.59) | 0.69 (0.02-1.37) |
