# Supplementary material for: Soil-transmitted helminthiasis in China: A national survey in 2014-2015
Source: PLoS Negl Trop Dis. 2021 Oct 19;15(10):e0009710. doi: 10.1371/journal.pntd.0009710 (PMC8555824; doi:10.1371/journal.pntd.0009710)
Supplement: S6 Table — (DOCX) [file pntd.0009710.s007.docx]

**S6 Table.** Estimated population infected of soil-transmitted helminthiasis by infection intensity and provinces in China in 2014-2015

| **Province** | **Hookworm infection** | | | **Ascariasis** | | | **Trichuriasis** | | |
| --- | --- | --- | --- | --- | --- | --- | --- | --- | --- |
|  | **Light** | **Moderate** | **Heavy** | **Light** | **Moderate** | **Heavy** | **Light** | **Moderate** | **Heavy** |
| **Beijing** | 0 | 0 | 0 | 845 | 575 | 0 | 291 | 110 | 0 |
| **Tianjin** | 0 | 0 | 0 | 609 | 81 | 0 | 0 | 0 | 0 |
| **Hebei** | 0 | 0 | 0 | 63307 | 0 | 0 | 0 | 0 | 0 |
| **Shanxi** | 196 | 0 | 0 | 97 | 0 | 0 | 508 | 0 | 0 |
| **Neimenggu** | 0 | 0 | 0 | 10661 | 1081 | 0 | 8089 | 111 | 0 |
| **Liaoning** | 0 | 0 | 0 | 32975 | 8669 | 444 | 2043 | 0 | 0 |
| **Jilin** | 0 | 0 | 0 | 36103 | 6190 | 0 | 1675 | 0 | 0 |
| **Heilongjiang** | 0 | 0 | 0 | 222 | 0 | 0 | 239 | 0 | 0 |
| **Shanghai** | 0 | 0 | 0 | 789 | 110 | 0 | 125 | 0 | 0 |
| **Jiangsu** | 34360 | 0 | 0 | 37331 | 356 | 0 | 3188 | 0 | 0 |
| **Zhejiang** | 238380 | 22800 | 11214 | 6915 | 572 | 0 | 9854 | 0 | 0 |
| **Anhui** | 1221265 | 44695 | 29604 | 15463 | 2235 | 0 | 125477 | 1233 | 0 |
| **Fujian** | 198616 | 13712 | 5843 | 4507 | 722 | 0 | 14465 | 1897 | 0 |
| **Jiangxi** | 783894 | 34334 | 39934 | 237268 | 60447 | 10940 | 270535 | 3047 | 0 |
| **Shandong** | 4362 | 0 | 0 | 53809 | 2011 | 1771 | 224067 | 22671 | 1771 |
| **Henan** | 62231 | 151 | 375 | 119604 | 173 | 0 | 88240 | 1245 | 0 |
| **Hubei** | 16369 | 0 | 116 | 37574 | 1057 | 0 | 64387 | 3357 | 0 |
| **Hunan** | 882650 | 43229 | 33675 | 569213 | 104927 | 10960 | 49455 | 5135 | 0 |
| **Guangdong** | 1690165 | 56504 | 14275 | 213676 | 30305 | 16226 | 80957 | 0 | 0 |
| **Guangxi** | 960678 | 26864 | 11986 | 362223 | 138074 | 8187 | 482119 | 14206 | 0 |
| **Hainan** | 334391 | 3612 | 8766 | 882 | 50 | 0 | 72576 | 109996 | 1514 |
| **Chongqing** | 642110 | 31460 | 26946 | 259209 | 47017 | 0 | 33376 | 599 | 0 |
| **Sichuan** | 6036096 | 653026 | 757754 | 1859530 | 1178801 | 456939 | 2629857 | 662397 | 0 |
| **Guizhou** | 814952 | 14963 | 26597 | 1041593 | 309242 | 31615 | 352396 | 5159 | 0 |
| **Yunnan** | 1095894 | 6978 | 35691 | 500336 | 143348 | 7715 | 1228296 | 14229 | 307 |
| **Xizang** | 369 | 0 | 0 | 22730 | 5155 | 0 | 5581 | 484 | 0 |
| **Shaanxi** | 0 | 0 | 0 | 239057 | 51407 | 31561 | 543 | 0 | 0 |
| **Gansu** | 0 | 0 | 0 | 278378 | 5113 | 773 | 718 | 0 | 0 |
| **Qinghai** | 0 | 0 | 0 | 24795 | 2801 | 0 | 0 | 0 | 0 |
| **Ningxia** | 2443 | 0 | 0 | 58688 | 346 | 219 | 1589 | 0 | 0 |
| **Xinjiang** | 0 | 0 | 0 | 59495 | 71 | 0 | 2048 | 0 | 0 |
| **Total** | 15019421 | 952328 | 1002776 | 6147884 | 2100936 | 577350 | 5752694 | 845876 | 3592 |
